# Genomic prediction of morphometric and colorimetric traits in Solanaceous fruits

**DOI:** 10.1093/hr/uhac072

**Published:** 2022-03-23

**Authors:** Hao Tong, Amol N Nankar, Jintao Liu, Velichka Todorova, Daniela Ganeva, Stanislava Grozeva, Ivanka Tringovska, Gancho Pasev, Vesela Radeva-Ivanova, Tsanko Gechev, Dimitrina Kostova, Zoran Nikoloski

**Affiliations:** 1 Center of Plant Systems Biology and Biotechnology, Plovdiv, 4000, Bulgaria; 2 Systems Biology and Mathematical Modeling, Max Planck Institute of Molecular Plant Physiology, Potsdam, 14476, Germany; 3 Bioinformatics, Institute of Biochemistry and Biology, University of Potsdam, Potsdam, 14476, Germany; 4 Maritsa Vegetable Crops Research Institute, Plovdiv, 4003, Bulgaria

## Abstract

Selection of high-performance lines with respect to traits of interest is a key step in plant breeding. Genomic prediction allows to determine the genomic estimated breeding values of unseen lines for trait of interest using genetic markers, e.g. single-nucleotide polymorphisms (SNPs), and machine learning approaches, which can therefore shorten breeding cycles, referring to genomic selection (GS). Here, we applied GS approaches in two populations of Solanaceous crops, i.e. tomato and pepper, to predict morphometric and colorimetric traits. The traits were measured by using scoring-based conventional descriptors (CDs) as well as by Tomato Analyzer (TA) tool using the longitudinally and latitudinally cut fruit images. The GS performance was assessed in cross-validations of classification-based and regression-based machine learning models for CD and TA traits, respectively. The results showed the usage of TA traits and tag SNPs provide a powerful combination to predict morphology and color-related traits of Solanaceous fruits. The highest predictability of 0.89 was achieved for fruit width in pepper, with an average predictability of 0.69 over all traits. The multi-trait GS models are of slightly better predictability than single-trait models for some colorimetric traits in pepper. While model validation performs poorly on wild tomato accessions, the usage as many as one accession per wild species in the training set can increase the transferability of models to unseen populations for some traits (e.g. fruit shape for which predictability in unseen scenario increased from zero to 0.6). Overall, GS approaches can assist the selection of high-performance Solanaceous fruits in crop breeding.

## Introduction

Solanaceae is one of the most important horticulture plant families [1]. The Solanaceous fruits, such as: tomato, pepper, and eggplant, are commonly consumed vegetables in human diet. Much effort has been put in cultivating Solanaceous crops to increase their fruit production, fruit quality, and disease resistance using different plant breeding technologies [2]. Moreover, Solanaceae family contains model species, including: tomato, potato, tobacco, and petunia, that have been employed to study plant developmental processes, genetic mechanisms of plant vegetative growth and reproduction as well as plant evolution [3, 4]. Further, extensive studies have begun to unravel the genetic regulation of Solanaceous fruit yield [5, 6] and quality [7–9].

Breeding has been revolutionized by developing machine learning models to accurately predict traits of interest [10]. Statistical modeling has also been instrumental in determining the genetic basis of traits by jointly analyzing phenotypic and genotypic data from diverse populations [11]. Therefore, these approaches can readily be used in breeding and determining the genetic basis of traits of Solanaceous fruits. A comprehensive description of phenotypic profiles is the initial step for characterizing and using of plant diversity as well as a basis for establishing new breeding programs [12, 13]. The morphometric and colorimetric traits of Solanaceous fruits include fruit size, shape, and color are conventionally described by the manual assignment of scores based on predefined categories [14], which are widely used for plant and fruit characterization [13, 15]. As a result, the conventional descriptors (CDs) of traits are prone to errors in scoring and are labor-intensive to acquire; in particular, in the case of closely related fruit traits or cultivar groups they are not so practical and precise. In contrast, Tomato Analyzer (TA) is a high-throughput fruit phenotyping tool that has been designed to characterize morphometric and colorimetric traits of Solanaceous fruits [16, 17]. It has been used in tomato [18–20], eggplant [21], and pepper [13, 22, 23] to investigate their phenotypic diversity and genetic basis. TA phenotyping system is more accurate than the CDs and can be used to quantify precise traits, such as fruit end-shape and asymmetry, which cannot be measured manually. Figàs et al. found the combination of CDs and TA provides a powerful tool for characterization and classification of tomato varieties, as well as for distinguishing between related cultivar groups [15].

The pivotal aim in plant breeding is to identify and select high-performance genotypes based on the traits of interest, such as fruit yield, fruit quality, and disease resistance. Marker-assisted selection (MAS) allows genotype selection via the genetic marker associated with the trait of interest [24]. The key step of MAS is to identify the associated markers (e.g. quantitative trait locus (QTL) mapping), which is labor-intensive and time-consuming. Moreover, MAS facilitates only the detection of the major effect genes. Genomic prediction is an advanced approach to estimate the genomic estimated breeding values using genetic markers, i.e. single-nucleotide polymorphisms (SNPs) [25]. Genomic prediction can be applied in selection of genotypes, in a process referred to genomic selection (GS). GS has been applied across different crops, including: maize [26], rice [27] and, recently, in tomato [28] and pepper [29] as an efficient way of artificial selection. The GS approach uses whole-genome SNPs so that the minor effect genes are also considered in the prediction. High predictability of valuable traits in unseen genotypes has the potential to reduce the breeding cycles and help to generate high-performance breeding lines that allow breeders to increase the genetic gain. Three categories of GS models were applied to plants and animals: regression-based, classification-based, and deep learning models, and the predictabilities can differ considerably between traits and models [10].

To comprehensively investigate the performance of GS models for morphometric and colorimetric traits in Solanaceous fruits, we collected the phenotypic and genotypic data from two Solanaceous crops, i.e. tomato and pepper. First, we applied the regression-based GS models using genome-wide SNPs and tag SNPs. We then employed multi-trait GS models to explore the predictabilities of correlated traits. Moreover, we also used the resulting models to predict the traits in an independent tomato population composed of wild tomato accessions. Finally, by using classification-based GS models to predict CD traits and compare the predictabilities in regression-based models of TA traits. The findings demonstrated that GS based on data of TA phenotyping system and tag SNPs can accurately predict the morphometric and colorimetric traits of Solanaceous fruits.

## Results

### Genomic selection of TA traits

The studied accessions were collected from 21 countries across the world for tomato and from six Balkan countries for pepper (Supplementary Table 1). The principal component analysis shows high genetic diversity of the employed tomato and pepper populations (Supplementary Figure 1; see Materials and methods). In total, 47 morphometric and colorimetric traits in ten trait groups were measured by Tomato Analyzer (TA) system using the same protocol in tomato and pepper populations (Supplementary Table 2; see Materials and methods) [20, 23, 30]. Trait group of basic measurement shows the highest heritability (0.95), followed by fruit shape (0.9) and fruit color (0.89) in tomato; the same findings are observed in pepper (Supplementary Table 2). Genetic markers were obtained from the tGBS sequencing (see Materials and methods). After quality control, filtering minor allele frequency (MAF) and less informative SNPs (see Materials and methods), there are 79 872 and 77 401 SNPs for usage in GS models in tomato and pepper collections, respectively.

**Figure 1:**
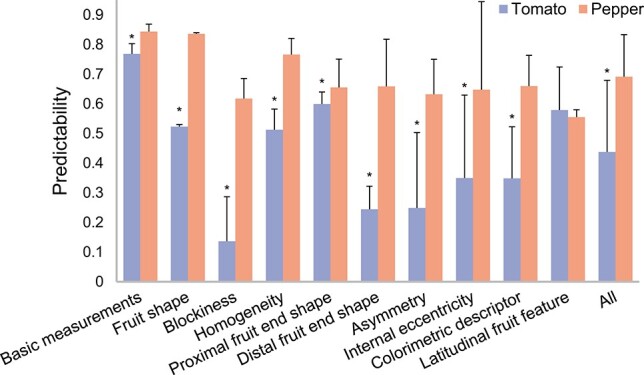
GS predictabilities in tomato and pepper. Predictabilities were assessed by the average Pearson correlation coefficient between measured and predicted trait using genome-wide SNPs with 150 cross-validations (i.e. 30 repetitions of five-fold cross-validation). Predictabilities are shown as mean values and standard deviations across all traits in the corresponding trait group. * denotes the significant differences between predictability of tomato and pepper in each trait group at level of 1 × 10^−3^ (two-sided paired t-test, n = 450 to 1350 cross-validations).

The state-of-the-art GS model, rrBLUP, is used to predict all TA traits and to assess the predictabilities in five-fold cross-validations in cultivated tomato and pepper populations (see Materials and methods). The average predictabilities, quantified by the Pearson correlation coefficients between measured and predicted traits, over all TA trait in pepper population (i.e. 0.69 ± 0.14) are higher than those for the same traits in tomato population (i.e. 0.44 ± 0.24) (Figure 1). Although the number of SNPs is comparable between tomato and pepper population, we observe 57.9% of increase in the average predictability of pepper compared to tomato. When evaluating the average predictabilities of each trait group, we found the GS performance of basic measurements and latitudinal feature groups, related to fruit size and weight, are similar between two Solanaceous crops. Interestingly, for the latitudinal feature group, the average predictability in pepper is lower than that in tomato. The other trait groups related to fruit shape and color are significantly different between the two Solanaceous species (p-value<1 × 10^−10^, paired t-test; Figure 1).

The predictabilities dramatically differ between ten trait groups in the two crops. The GS models for basic measurements group shows the best prediction performance (average predictability of 0.77), while the blockiness group performs the worst (0.14) in tomato (Figure 1, Supplementary Table 3). These results are consistence with heritability that basic measurement group shows highest heritability (0.95) and blockiness group shows relatively low heritability (0.54) (Supplementary Table 2). In pepper, the best prediction trait group is basic measurements (0.844), followed by fruit shape (0.836), while the latitudinal feature group shows the worst predictability (0.56). The heritability of basic measurement and fruit shape group is the highest (both of 0.95) in pepper indicates the better prediction performance is for high heritability traits. Particularly, the predictabilities of fruit size and shape descriptors are higher than the more complex and detailed descriptors (e.g. fruit color, end shape, asymmetry) in both crops. For the individual traits, the best prediction is for fruit perimeter (predictability of 0.81) in tomato and width at middle height (0.89) in pepper, which are both part of basic measurements (Supplementary Table 4). The worst prediction is found for proximal eccentricity in internal eccentricity group in both tomato (−0.09) and pepper (0.21). These results implied the basic traits, e.g. fruit size, can be easily predicted from genomic information, while the detailed traits, e.g. eccentricity, are more complex and less powerful to be predicted from genomic markers.

### Predictabilities using tag SNPs

**Figure 2:**
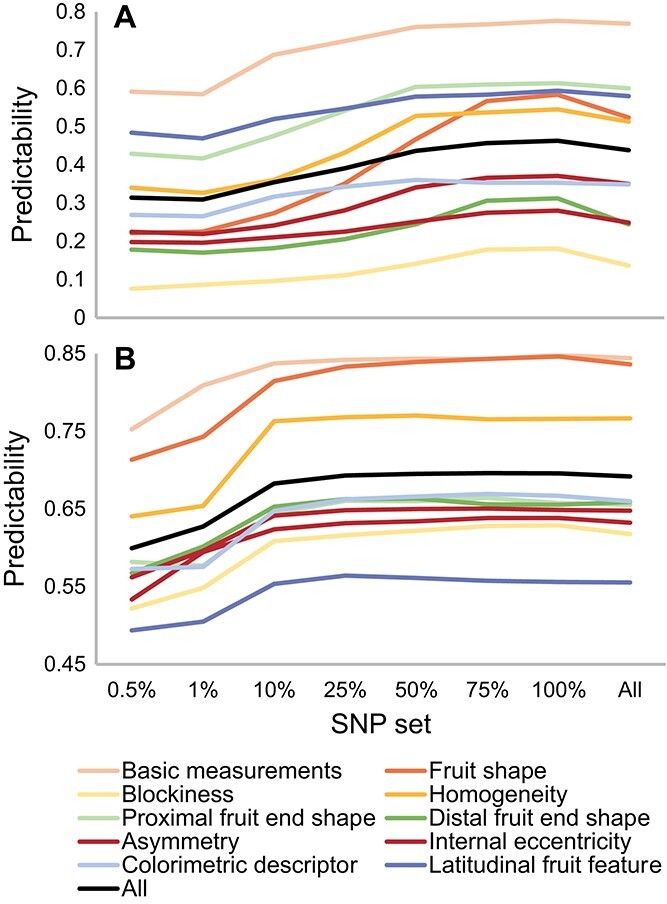
GS predictabilities using tag SNP. Predictabilities are shown as TA trait groups in tomato (A) and pepper (B). The x-axis indicates the percentage among all independent tag SNPs as well as genome-wide SNPs used as predictors in the GS model. Predictabilities were assessed by the average Pearson correlation coefficient between measured and predicted trait with 150 cross-validations (i.e. 30 repetitions of five-fold cross-validation).

To reduce the computation complexity as well as to investigate the effect of SNP number on GS predictability, the tag SNPs – the most informative SNPs across genome based on linkage disequilibrium [31] – were selected and used as predictors in GS models. To the end, 13 590 SNPs were determined as tag SNPs in tomato, which account for 17% of genome-wide SNPs, and 19 685 tag SNPs were selected in pepper, equivalent to 25.4% of genome-wide SNPs. The average predictabilities of all TA trait groups using tag SNPs are approximately equal to the one by using genome-wide SNPs in the two crops. Surprisingly, for some trait groups, the average predictabilities using tag SNPs are higher than using genome-wide SNPs, for instance, fruit shape and distal end shape increase by 11.4% and 27.6% in tomato, respectively (Figure 2).

For further exploration of the effect of SNP number, six subset scenarios in different percentages of tag SNPs as the most representatives were selected (see Materials and methods). The average predictabilities are increasing with the number of tag SNPs for all trait groups in both tomato and pepper (Figure 2). Interestingly, the predictability of fruit shape group in tomato increases from 0.22, using 68 tag SNPs (0.5% of all tag SNPs), to 0.58 when using all tag SNPs (Supplementary Tables 5 and 6). This result indicates that a large number of genes might be involved in the control of fruit shape in tomato. In contrast, the basic measurement of fruit size in pepper only increases by 12.7% (from 0.75 to 0.85) by increasing the tag SNPs number from 98 to 19 685, which infers few major genes might affect the traits. Considering both the computation load and prediction performance, the tag SNPs set at the predictability reached the plateau (25% of all tag SNPs; 3398 SNPs in tomato and 4921 SNPs in pepper) were kept for further analysis.

### Genomic selection of multiple traits

The phenotypes in each trait group describe one aspect of Solanaceous fruit characteristics. The genomic prediction could be extended to multiple traits to consider their relationships. The correlation coefficients between traits were firstly calculated within each trait group. In the basic measurement group, the average correlation coefficient is 0.83 in tomato, the highest correlation is between width at middle height and maximum width (r = 0.99), and the lowest is between width at middle height and height at middle width (r = 0.5) (Supplementary Figure 2A). In pepper, the correlations within basic measurement group differ to those in tomato, where the width at middle height is negatively correlated to three height-related traits, and maximum width shows no correlations to fruit height (Supplementary Figure 2B). The correlation coefficients between traits in the colorimetric descriptor group are relatively low. Particularly, for the three traits, i.e. average red, green and blue, the average correlation coefficient is 0.54 and 0.62 in tomato and pepper, respectively (Supplementary Figure 2C, D). After the color space transformation, the average absolute correlation coefficient between the corresponding traits (i.e. average *L*, *a* and *b* value) is 0.47 in tomato, in which only the correlation coefficient between the average *L* and *b* value is positive (r = 0.72) while the other two are negatively correlated. For the three fruit shape-related traits, all correlations are relatively high (averaged r = 0.99 in tomato, r = 0.93 in pepper). The trait pericarp area and thickness are highly negative correlated (r = −0.997 in tomato, r = −0.84 in pepper, Supplementary Figure 2A, B).

**Figure 3:**
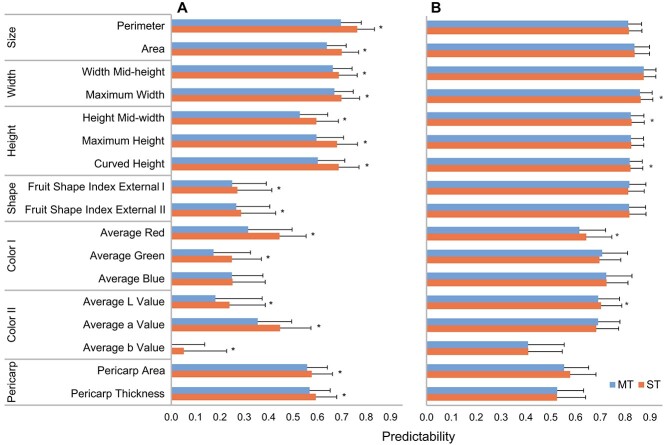
GS predictabilities of multi-trait model. Seven multiple trait groups, i.e. fruit size, width, height, shape, color based on RGB and CIELab as well as pericarp were tested using both single trait (ST) and multiple trait (MT) GS models in tomato (A) and pepper (B). Predictabilities were assessed by the average Pearson correlation coefficient between measured and predicted trait with 150 cross-validations (i.e. 30 repetitions of five-fold cross-validation). * denotes the significant differences between the predictability of ST and MT GS model at level of 1 × 10^−4^ (two-sided paired t-test, n = 150 cross-validations).

The multi-trait GS model is the extension of single-trait linear mixed model, where the response is composed of multiple variables (see Materials and methods). The results show that there is no considerable improvement in predictabilities using multi-trait GS model comparing to single-trait GS model in all testing scenarios (Figure 3). Most of traits show significantly better prediction based on the single-trait GS model comparing to multi-trait models in tomato. For the trait width at middle height, fruit shape index external I and pericarp thickness as well as average green and *a* value, the multi-trait GS models perform slightly better than single-trait GS model in pepper (Figure 3). The Rv coefficients, which evaluate the correlations of multiple trait matrices, show relatively higher predictabilities in pepper than tomato, especially for fruit shape and color (Supplementary Table 7). The findings indicate that multi-trait GS could be an alternative model to predict some agronomic traits.

### Predictions for a population of wild tomato

**Figure 4:**
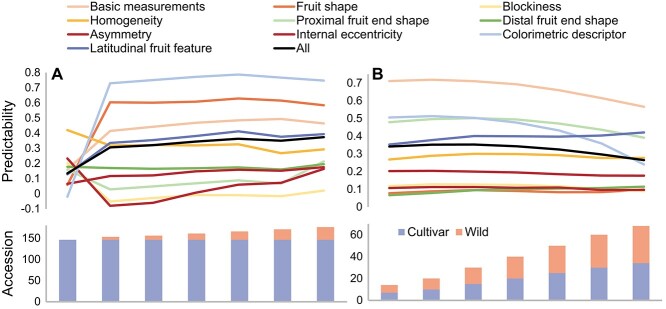
Prediction of wild tomato accessions. Scenarios of different number of wild tomato accessions together with all cultivated accessions in the training set and test on the remaining wild accessions (A). Scenarios of different number of wild tomato accessions together with the same number of cultivated accessions in the training set and test on the remaining accessions (B). Predictabilities were assessed by the average Pearson correlation coefficient between measured and predicted trait in testing set with 150 cross-validations (i.e. 30 repetitions of five-fold cross-validation).

To validate GS process and mimic the practical scenario in plant breeding, an independent population was applied to test the GS model performance. The population consists of 34 wild tomato accessions from seven species that both genotypic and phenotypic data are available (see Materials and methods). The principal component analysis and polygenic tree show that these wild accessions are genetically distant to cultivated accessions (Supplementary Figure 3; see Materials and methods). The GS models were firstly trained on all cultivated tomato accessions and assessed the predictabilities in all wild tomato accessions. The average predictability over all TA trait is 0.14, the predictability is only 0.16 for basic measurement group. Unexpectedly, fruit shape and color show the worst performance (Supplementary Table 8). The possible reason could be the distant relationship between the training (cultivated tomato accessions) and testing population (wild tomato accessions), which was known as a key determinant of the GS model performance. To check the effects of relationship between training and testing population on predictability of GS models, six scenarios were investigated for different number of wild accessions used to train GS models and the remaining used as testing set (see Materials and methods). When only one sample in each wild species (i.e. seven accessions) were added in training set, the predictabilities show dramatic changes in three directions (Figure 4A). The predictabilities rise rapidly from nearly zero to relatively high values for fruit shape (from 0.06 to 0.6) and colorimetric descriptor (from −0.02 to 0.73) (Supplementary Table 8). The predictabilities for basic measurements, internal eccentricity and latitudinal feature increase by ~one-fold. However, the predictability of four trait groups, such as blockiness and proximal end shape, show a decrease by adding wild tomato accessions in training set. Additionally, the predictability of distal end shape is the same for training set with/without wild accessions.

When adding more wild accessions (i.e. 10, 15, 20, 25, 30) in training set, the predictabilities show relatively modest increase (Figure 4A). Comparing the scenarios of seven and 30 wild accessions in the training set, we observed an average increase of 22.1% across all TA trait. The predictability of internal eccentricity increases by 50.2% (from 0.12 to 0.18), and basic measurement by 11.9% (from 0.41 to 0.46). Interestingly, the GS predictability for fruit shape and homogeneity show slightly decrease (from 0.6 to 0.58 and from 0.32 to 0.29, respectively, Supplementary Table 8). The most decrease trait is homogeneity in rectangular which is from 0.22 to 0.12 (Supplementary Table 9). Another scenario is to balance the number of cultivated and wild accessions in training set (see Materials and methods). The basic measurement, proximal fruit end shape and colorimetric descriptor show decrease as more accessions included in the training set, while the others remain almost unchanged (Figure 4B, Supplementary Table 8). These findings indicate the importance of relationship between training and testing population and the number of distant samples did not have a large effect on GS predictability. Our results showed that only few samples (i.e. one accession in each species) from the distant population could represent the population structure and result in good performance of GS models.

**Figure 5:**
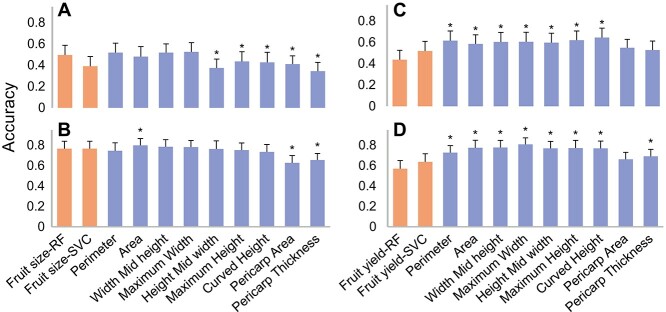
Comparison of predictabilities between CD and TA traits. The TA trait predictabilities were calculated based on the same class proportion of CD trait, i.e. fruit size (A, B) and fruit yield per plant (C, D) in tomato and pepper, respectively. Predictabilities were assessed by the average accuracy coefficient with 150 cross-validations (i.e. 30 repetitions of five-fold cross-validation). * denotes the significant differences between the highest predictability model of CD trait and each TA trait at level of 1 × 10^−4^ (two-sided paired t-test, n = 150 cross-validations).

### Prediction of traits scored by conventional descriptors

Tomato Analyzer phenotyping system in comparison to conventional descriptor system can measure the corresponding traits more accurately and needs less manual work. To evaluate the predictability of CD traits in categories, two classification-based GS models, random forest and support vector classification, were applied (see Materials and methods). Two CD traits, fruit size and fruit yield per plant, were analyzed and compared with seven related TA traits of fruit perimeter, area, width, height as well as pericarp area and thickness. The average correlation coefficients between fruit size and studied TA traits are higher than the ones between fruit yield and TA traits in two Solanaceous crops. The average correlation coefficient between fruit size and TA traits is 0.64 in tomato, while the correlation coefficient between fruit size and pericarp thickness is negative (r = −0.64), and is positive for all other traits in tomato (Supplementary Figure 4A). Similar correlations were observed in pepper (Supplementary Figure 4B).

The average predictability assessed in cross-validations by area under curve (AUC) coefficient of random forest model (0.76) is higher than the one from the support vector classification model (0.7) across all analyzed scenarios (Supplementary Table 10). To compare the predictabilities between TA and CD traits, the same assessment approach using accuracy coefficient was applied to the predictions from regression- and classification-based GS models (see Materials and methods). In comparison to CD trait of fruit size, the largest predictability among the two classification-based GS models is similar to those of fruit perimeter and width TA traits, but significantly higher than those of pericarp area and thickness TA traits in two Solanaceous crops (p-value<1 × 10^−4^, paired t-test; Figure 5A, B). Among all CD and TA traits, the highest predictability is for maximum width and 6% higher than fruit size in tomato and is for fruit area with 4% increase in pepper. In the comparison scenario of CD trait of fruit yield per plant, the predictabilities of all TA traits except pericarp traits are significantly higher than the CD trait in the two Solanaceous crops (p-value<1 × 10^−4^, paired t-test; Figure 5C, D). The results indicate the usage of TA phenotyping system is better than CD phenotyping system in respect of predictions of fruit size and yield-related traits in Solanaceous crops.

## Discussion

Our extensive GS modeling work showed that the predictabilities of the same traits may differ between two Solanaceous crops, tomato and pepper. It remains to be investigated whether similar predictabilities for the traits e.g. fruit size and weight are due to same (or similar) genetic architecture and the predictabilities of traits e.g. fruit shape and color which are species-specific due to the different molecular mechanisms. The genetic effects can be partly predicted by using the whole-genome markers, while the remaining effects may be explained by other factors, including: post-transcriptional regulation, epigenetic regulation, and environment.

The tag SNPs lead to GS models with best performance. Previous studies showed the predictabilities would not increase significantly after a threshold of SNP number [32]. Improvement in trait predictability using tag SNPs in this study could be explained by the removal of the effect that redundant SNPs have on the computational problem solved (i.e. rrBLUP). Instead of whole-genome sequencing, genotyping only tag SNPs provides a more efficient way to predict traits due to reduction in costs and may lead to increase in performance of GS models. Another way to select the tag SNPs is based on the predefined haplotype blocks [33]. The representatives of haplotype blocks can be assigned as tag SNPs. Moreover, the haplotype alleles can be coded as multiple levels variables, comparing to the standard, three-level genotype allele. The haplotype alleles as predictors in GS models have been studied previously and shown improvement in the predictability comparing with three-level SNP coding data set [34].

In the context of machine learning, one of the key determinants of predictability is the relationship between training and testing data set. Similar to previous studies, we observed that it is crucial to increase the number of accessions in the training data for more accurate predictions of unseen accessions [3]. When considering the relationship between training and testing set, we observed that the consideration of accessions from very different populations lead to significant improvement of predictabilities, and by adding just one accession in each species, the predictability increased dramatically in our study. The findings showed the diverse germplasm collection will contribute to the crop breeding in Solanaceous fruits.

The new technologies for plant phenotyping are developing rapidly, e.g. using scanner to record morphometric and physiological traits [35]. In our study, fruit phenotypic diversity examined by CDs and image-based high-throughput TA provided an comparative efficacy assessment of respective phenotyping methods where TA fruit phenotyping resulted in better trait predictability compared to CDs. To improve the genetic gain, our study of trait predictability using comparative assessment between TA and CDs would allow breeders to make well-informed decisions than just using either TA or CDs [20, 23]. Meanwhile, the investigation on the number of traits, i.e. multi-trait GS models, showed no improvement in our work. Previous studies showed that the multi-trait model yielded similar predictabilities comparing to single-trait GS model, while improvement was observed particularly for low-heritability traits [36] or in cross-validations whereby a related trait was measured in testing population [37]. More accurate measurements of traits of interest that exhibit natural variability could facilitate new developments in plant breeding and assist breeders to increase the genetic gain. Overall, the findings indicate the importance of germplasm and trait diversity in the GS approach in Solanaceous fruits.

## Materials and methods

### Plant materials

For tomato data set, a panel of 192 accessions was collected across the world, in which 149 accessions are *Solanum lycopersicum*. The remaining 43 accessions are wild species, including 12 *Solanum pimpinellifolium*, 11 *Solanum habrochaites*, nine *S. peruvianum*, three *S. chilense*, three *Solanum chmielewskii*, two *Solanum corneliomulleri*, one *Solanum arcanum*, one *Solanum cheesmaniae* and one *Solanum pennellii* (Supplementary Table 1). The seeds were sown in greenhouse at the end of March 2018, and five-week old tomato seedlings were transplanted in open field in a two-row planting scheme Each accession was represented by ten plants with four independent trials of randomized complete block design.

For pepper data set, a panel of 180 *Capsicum sp.* accessions were collected mainly from the Balkan region, in which 177 are *C. annuum*, two *C. frutescens* and one *Capsicum baccatum* (Supplementary Table 1). The seeds were sown in greenhouse in March 2018, and six-week old pepper seedlings were transplanted to open field. The same trial design as tomato was applied in pepper, but in three independent trials. All accessions were grown in accordance with adopted technology for mid-early field production. The fertilization, irrigation, plant protection, and microclimate were the same for all accessions. The fruits were harvested at the 9–10 and 7–10 week after flowering for tomato and pepper, respectively. Tomato fruits were harvested at full ripening stage. Pepper fruits were harvested in accordance with their usage: pungent and sweet green genotypes – before maturity at intermediate stage of ripening, while others were harvested at full ripening stage.

### Phenotype collections

To collect the fruit morphometric and colorimetric traits in both tomato and pepper, eight fruits replicates per trial were sampled. The phenotypes were preliminarily measured by the CDs based on a previously established scoring system. Each trait was pre-defined by three to ten categories, and the fruits were assigned to the corresponding category manually. In total, 22 conventional descriptors were collected for traits including: fruit size in five categories (very small, small, medium, big, very big), fruit yield per plant in five categories (very low, low, medium, high, very high). To measure the phenotypes using the TA system, four of the samples were cut longitudinally and four were cut latitudinally. The fruits were scanned with an Epson Perfection v19 J371A photo scanner (Epson, Amsterdam, The Netherlands) at a resolution of 300 dpi. Image data of each accession were subjected to morphometric and colorimetric analysis using TA software (version 3) [38, 39]. A total of 47 different fruit morphometric and colorimetric traits were measured from the longitudinally and latitudinally cut fruits. In the longitudinal section, the traits were measured across nine trait groups including fruit basic measurements, fruit shape, blockiness, homogeneity, proximal fruit end, distal fruit end, asymmetry, internal eccentricity, and fruit color. Three traits, i.e. lobedness degree, pericarp area and pericarp thickness, related to internal fruit features (i.e. pericarp, placenta, and septum) were measured in latitudinal-cut images. All parameters in TA software were set the same as used in the previous studies [16, 40].

For each TA trait, a linear mixed model was applied to estimate the genotype effect among trials in both tomato and pepper. The average value of all replicates in each trial were calculated, then used to fit the linear mixed models. The intercept was set as fixed effect, genotype and trial effects were set as random effect in the models. The variance components and best linear unbiased predictions (BLUPs) of random effects were calculated using R package lme4 [41]. Heritability was calculated as the ratio between the variance of genotype and total variance in each studied trait. The summations of fixed effect, i.e. intercept, and BLUP values of genotype effect of each accession were used as the phenotypic value in the GS modelling.

### Genotype sequencing

The leaf tissue was sampled from a single plant at ~four weeks after planting. Collected material was lyophilized before DNA extraction according to standard protocol. Samples were quality controlled prior to tGBS library construction and each sample was then used to construct one or more sequencing libraries. All samples were genotyped using tGBS technology with the restriction enzyme *Bsp1286I* using the Illumina HiSeq X. Raw sequence data was debarcoded and reads were assigned back to their corresponding samples before performing quality trimming to remove low-quality regions at the beginning and end of each read. Considering both crops to be sequenced, we first processed the tomato collection followed by pepper collection. In tomato, trimmed reads were aligned to *S. lycopersicum* v4.0 reference genome of tomato (ftp://ftp.solgenomics.net/tomato_genome/assembly/build_4.00). SNP calling was conducted using only those reads that align to a single location in the reference genome. Quality control of missingness rate applied to raw data and the missing SNPs were imputed using Beagle v4.1 [42] with 50 phasing iterations and other default parameters. The raw data consisted of 415 959 SNPs. After filtering at most 50% missingness, the number of reliable SNPs was 281 558. Further filtering of SNPs with minor allele frequency (MAF) less than 5% was applied, resulting in 122 222 SNPs in the genotypic data set. To reduce the computational intensity of GS models, the less informative SNPs, i.e. high linkage disequilibrium (LD) SNPs, located distant on chromosomes due to population structure, were excluded using the LD filter of 0.999. To the end, a total of 79 872 SNPs and 180 successful phenotyped and genotyped accessions including 34 wild species were considered in GS models of tomato.

In pepper, a similar procedure was applied as implemented in tomato. Available genome sequence of *Capsicum annuum* v1.6 was used as a reference genome (http://peppergenome.snu.ac.kr/download.php). Number of raw data and reliable data filtering with missingness was 1 149 249 and 536 934 SNPs, respectively. After filtering with MAF less than 5%, 114 154 SNPs were retained. By removing the less informative SNPs and overlapped with available phenotyped accessions, a total of 77 401 SNPs from 162 accessions were used in GS models of pepper.

### Population structure analysis

Principal component analysis (PCA) was applied to analyze the population structure of studied tomato and pepper collections. The SNP data was projected using PCA to a number of independent principal components (PCs). The first and second PCs were used to illustrate the genetic variation between accessions. The phylogenetic tree of cultivarted and wild tomato accessions were calculated based on the SNP data in MEGA using all default parameters [43]. The illustration of phylogenetic tree was by the iTOL online tool [44].

### Tag SNP selection

The tag SNP was selected based on the linkage disequilibrium of genome-wide SNPs using the software *FastTagger* [45]. The tagger rules were built on the co-occurrences of the alleles of SNPs by the MMTagger algorithm. The SNP data set were firstly selected for all tag SNPs using the default parameters on each chromosome. To investigate the different SNP number scenarios, we used the algorithm to select a given number of the most informative tag SNP. Different percentages, i.e. 0.5%, 1%, 10%, 25%, 50% and 75%, of the number of all tag SNPs, were selected and assigned as tag SNP subsets. The tag SNP subsets were treated as predictors to investigate the effect of SNP numbers on the performance of GS models.

### Regression-based GS model

For TA traits, the state-of-the-art GS model in plant breeding, the ridge regression best linear unbiased prediction (rrBLUP) was applied [46]. The rrBLUP model was a mixed linear model, given by

$$ y= Xb+ Zu+e, $$

where $y$ is a vector of phenotype, $X$ is the fixed effect design matrix of identity matrix, $b$ is the fixed effect of intercept, $Z$ is a matrix of genetic markers, $u$ is the marker effect as random effect and $e$ is the residual error. This approach can shrink all effects toward zero equally, corresponding to the assumption that all markers have a common variance. All models were implemented using the R package *rrBLUP* [47].

### Multi-trait GS model

To explore the correlation between traits, particularly within each trait group, and the predictabilities of multiple traits in GS models, seven multiple trait scenarios were considered. Four scenarios corresponded to two-trait GS models: perimeter and area, width at middle height and maximum height, fruit shape index external I and II, and pericarp area and pericarp thickness; three scenarios considered three-trait GS models: average red, green, blue value, average *L*, *a*, *b* value, and maximum height, height at middle width, curved height. The multi-variate linear mixed model was solved with the same procedure as the univariate linear mixed model by considering the respective set of traits in the response of the linear mixed model. The multi-trait GS model considered the covariance of traits in the group into the variance matrix of random effects. The multi-variate models were implemented and solved using R package *sommer* [48]. The predictabilities of multi-trait GS models were evaluated for the individual trait (see Predictability assessment) as well as multiple trait matrix using Rv coefficient in each scenario [49].

### Classification-based GS model

For CD traits, two classification models, random forest (RF) and support vector classification (SVC) were utilized to predict the categories of each trait of interest. All classification-based GS models were implemented in R package *caret* [50]. For the RF model, the number of variables sampled as candidates was set to the root square of number of SNPs as default and the number of trees set to 1000. For the SVC model, 15 arbitrary values were selected to fit the parameters C and $\sigma$. All parameters were selected by cross-validations using the metric of accuracy.

### Predictability assessment

To assess the performance of the GS models, cross-validation schemes were applied for each trait. The accessions were split into five equal-size folds randomly; one fold was used as testing data and the remaining four folds were used as training data in each cycle. The cross-validations were performed 30 times, resulting in 150 cross-validation sets. In each set, four coefficients, Pearson correlation, Spearman correlation, matched rate of top 30% and 15% accessions, were used to assess the predictability in regression-based models. For the classification-based models, the predictabilities were evaluated by two coefficients, the accuracy and area under curve (AUC). Accuracy was defined by the ratio between number of true positive and true negative samples and all samples; AUC is the area under receiver operating characteristic (ROC) curve which is the true positive rate against the false positive rate. To learn the classification model in a balance scenario, synthetic minority over-sampling technique (SMOTE) method was applied to address the unbalance between classes in the data set [51]. For the purpose of comparing the predictabilities between regression- and classification-based models, the predicted TA traits from regression model were assigned to categories in the same quantiles of related CD traits. The coefficient of accuracy can be applied to the classified regression predictions and can be compared in the same manner in classification-based GS models.

When evaluating the predictabilities of wild tomato species, a certain number of wild accessions were randomly selected from all wild accessions in 150 cross-validations. The numbers of the wild accessions were assumed to be enumerated in a grid sequence (i.e. 7, 10, 15, 20, 25, 30) in scenarios in which ensure at least one accession of each wild species in the training set. These wild accessions were used together with all domesticated tomato accessions as training data set in GS models then tested on all remaining wild accessions. In addition, the balanced scenarios of which the number of cultivated and wild accessions (i.e. 7, 10, 15, 20, 25, 30, 34) were the same in training set, and evaluated on all remaining accessions. The random selections were imposed on each scenario independently. The predictabilities were assessed the same as the regression-based GS model.

## Acknowledgements

This research was supported by the European Union’s Horizon 2020 research and innovation programme, project PlantaSYST (SGA-CSA No. 739582 under FPA No. 664620), as well as the BG05M2OP001-1.003-001-C01 project, financed by the European Regional Development Fund through the Bulgarian “Science and Education for Smart Growth” Operational Programme.

## Author Contributions

HT, ZN, DK designed the project, VT, DG, SG designed the field experiments and collected the conventional description data, IT, SG, ANN collected the Tomato Analyzer data, DK, TG, GP, VRI prepared for sequencing, HT, JL analysed the data, HT, ZN interpreted the results, HT, ZN wrote the manuscript. All authors edited and approved the final manuscript.

## Data availability

The datasets and code in this study are available in the UTL: https://github.com/Hao-Tong/GS_Solanaceae.

## Conflicts of interests

The authors declare no conflicts of interest.

## Supplementary data

Supplementary data is available at *Horticulture Research* online.

## Supplementary Material

Web_Material_uhac072
