# Supplementary material for: Genomic prediction of morphometric and colorimetric traits in Solanaceous fruits
Source: Hortic Res. 2022 Mar 23;9:uhac072. doi: 10.1093/hr/uhac072 (PMC9157653; doi:10.1093/hr/uhac072)
Supplement: Web_Material_uhac072 [file web_material_uhac072.zip › Supplementary Information.docx]

**Genomic prediction of** **morphometric and colorimetric traits in** **Solanaceous fruits**

Tong, H., Nankar, A.N., Liu, J. et al.

**Supplementary Figures**

**
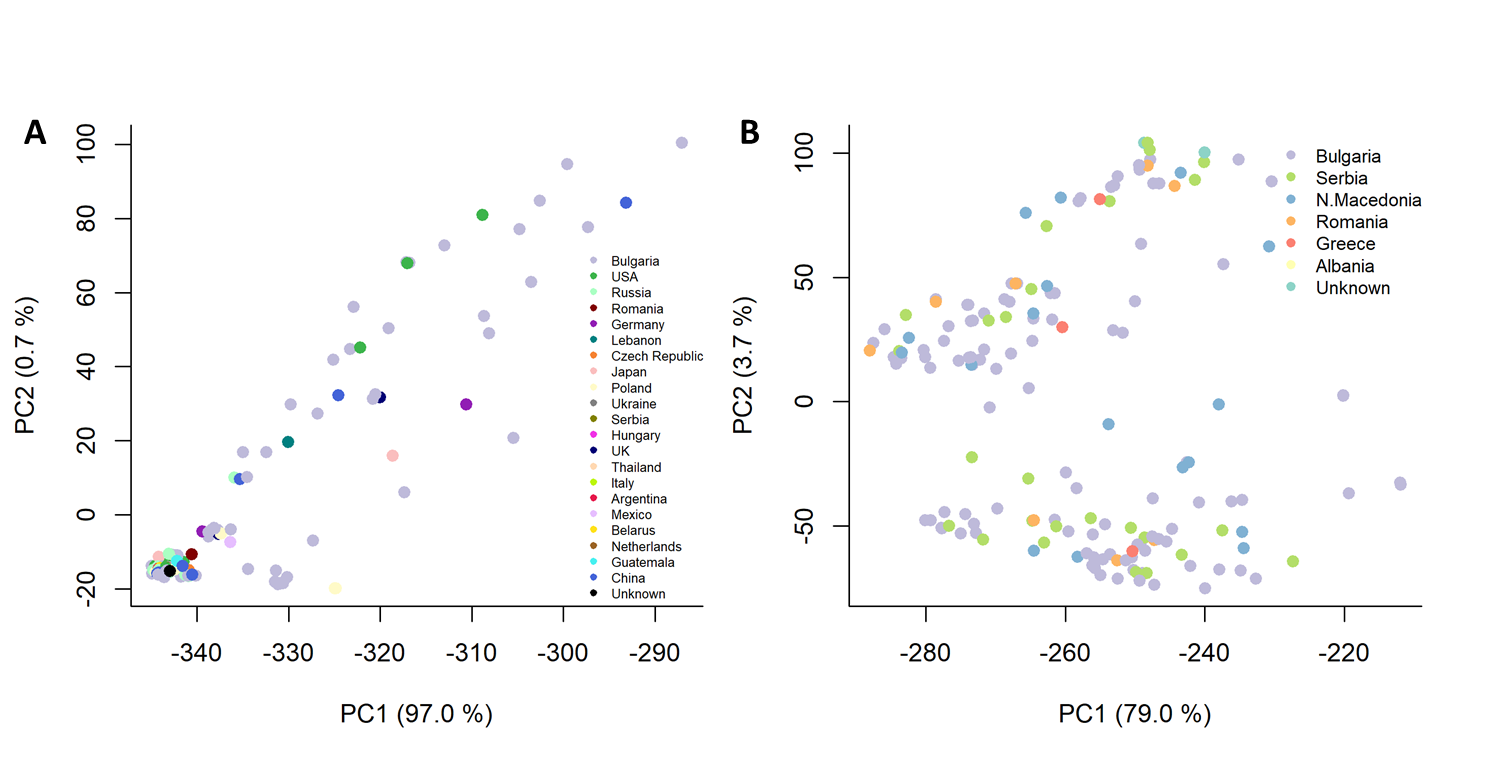
**

**Supplementary Figure 1. Population structure of tomato (A) and pepper (B) accessions.** The first two principal components from the principal component analysis using genome-wide SNPs are shown.

**
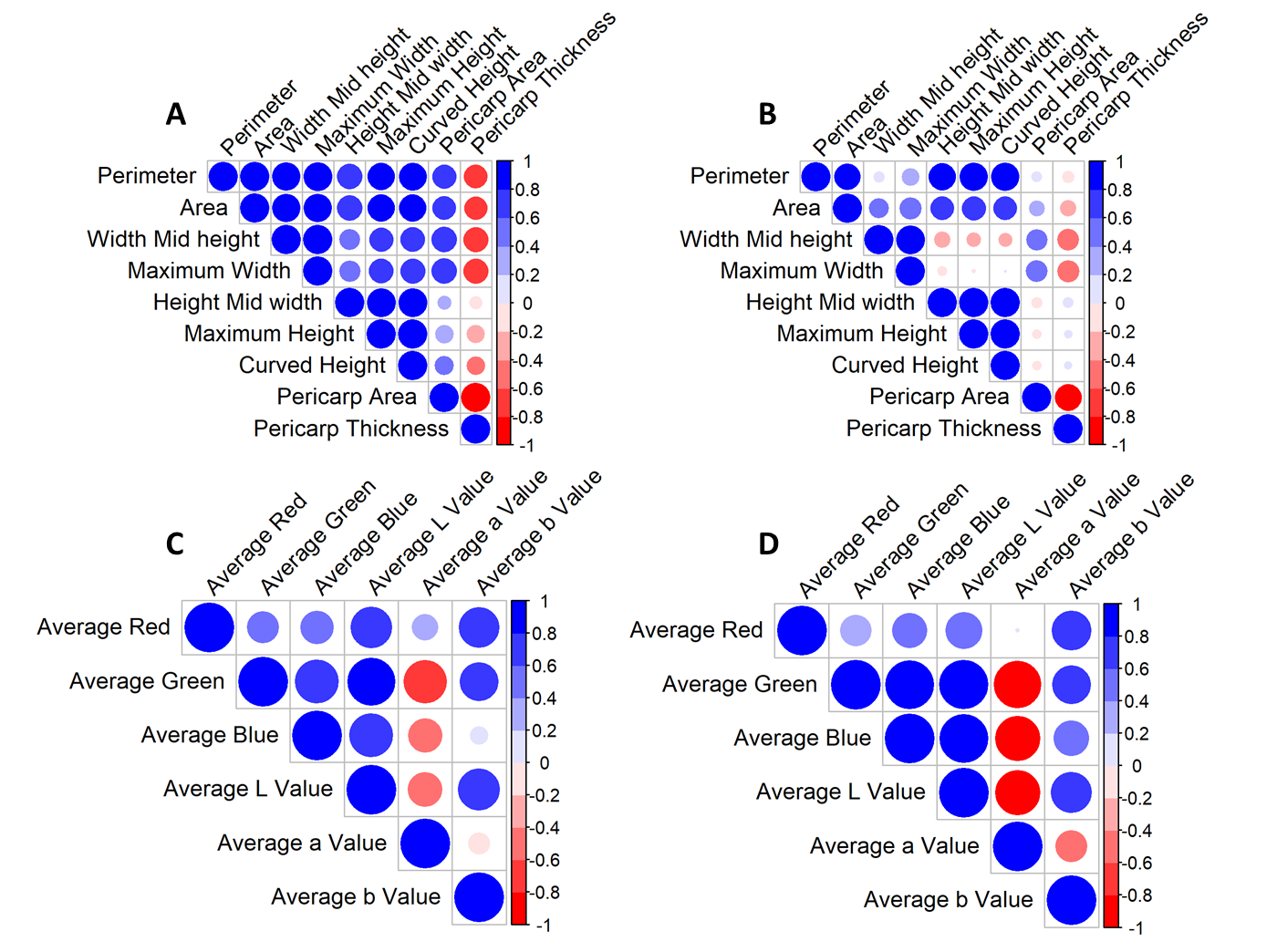
**

**Supplementary Figure 2. Correlations between TA traits in trait group.** Correlation coefficients are shown between traits related to fruit size (A, B) and fruit color (C, D) in tomato and pepper, respectively.

**
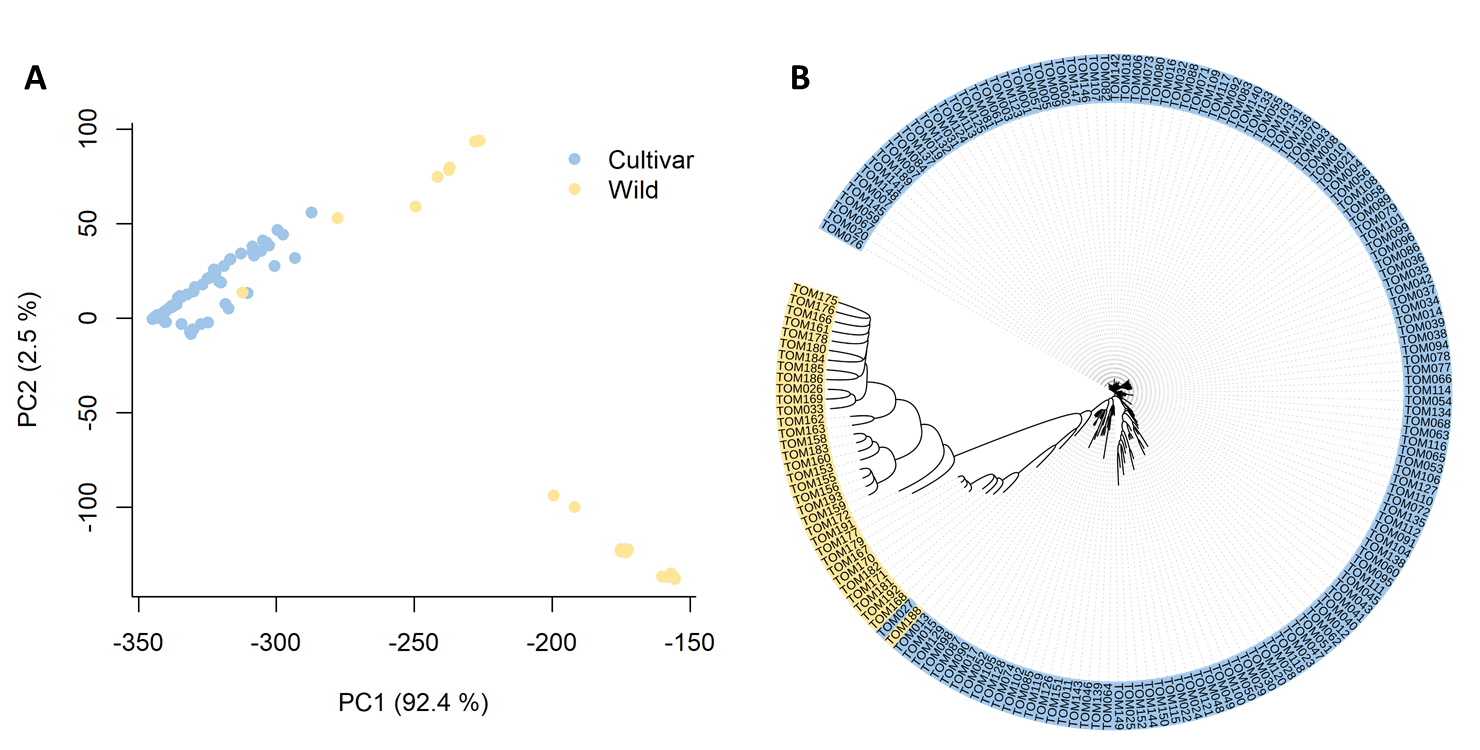
**

**Supplementary Figure 3. Population structure of cultivated and wild tomato accessions.** The first two principal components from the principal component analysis (A) and phylogenetic tree (B) using genome-wide SNPs are shown.


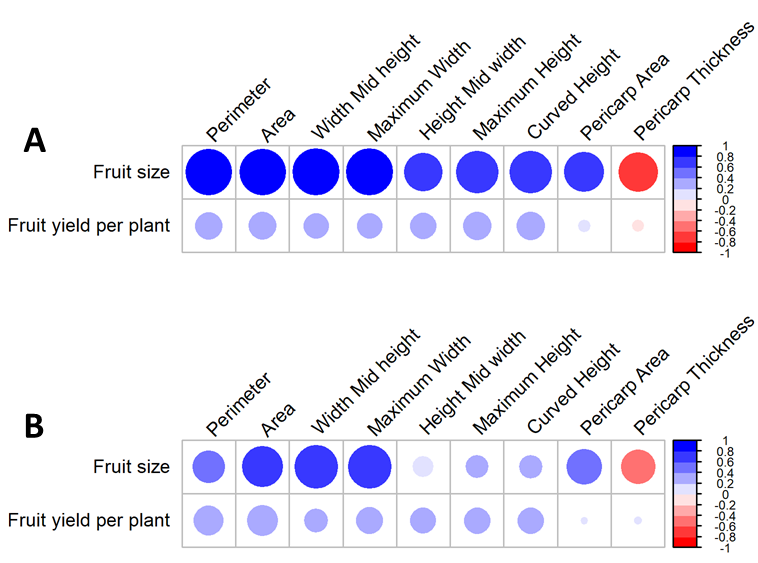


**Supplementary Figure 4. Correlations between CD and TA traits.** Correlation coefficients are shown between two CD traits, fruit size and fruit yield per plant, and related TA traits in tomato (A) and pepper (B), respectively.

**Supplementary Table Captions**

**Supplementary Table 1.** List of tomato and pepper accessions used in this study.

**Supplementary Table 2.** Tomato Analyzer traits and corresponding trait groups. The raw data of three (pepper) or four (tomato) independent trails as well as the heritability and BLUP value from linear mixed model are shown in separate sheets.

**Supplementary Table 3.** Genomic selection predictabilities of TA trait groups using genome-wide SNPs. The values in the table showed the averaged predictabilities evaluated in cross-validations and corresponding standard deviation over all traits in each group.

**Supplementary Table 4.** Genomic selection predictabilities of TA traits using genome-wide SNPs. Genomic selection predictabilities evaluated in cross-validations.

**Supplementary Table 5.** Averaged genomic selection predictabilities and standard deviation of TA trait groups using tag SNPs. The percentage indicated the proportion of tag SNPs used as predictors in GS model, and the first row showed the corresponding number of tag SNPs. Genomic selection predictabilities evaluated using the Pearson correlation coefficient between predicted and measured values in testing set in cross-validations.

**Supplementary Table 6.** Genomic selection predictabilities of TA traits using tag SNPs. The percentage indicated the proportion of tag SNPs used as predictors in GS model, and the first row showed the corresponding number of tag SNPs. Genomic selection predictabilities evaluated using the Pearson correlation coefficient between predicted and measured values in testing set in cross-validations.

**Supplementary Table 7.** Genomic selection predictabilities of multi-traits GS model. Genomic selection predictabilities evaluated in cross-validations and the Rv coefficients indicated the correlations of multi-trait matrices between prediction and measurement in testing set.

**Supplementary Table 8.** Averaged genomic selection predictabilities and standard deviation of TA trait groups in wild tomato population. Seven scenarios of different number of wild tomato accessions together with all cultivated accessions assigned in the training set and test on the remaining wild accessions were investigated (Scenario A). Seven scenarios of different number of wild tomato accessions together with the same number of cultivated accessions in the training set and test on the remaining accessions (Scenario B). The values of column names indicated the number of wild tomato accessions used to train the GS model. Genomic selection predictabilities evaluated using the Pearson correlation coefficient between predicted and measured values in testing set, i.e. the remaining tomato accessions, in cross-validations.

**Supplementary Table 9.** Genomic selection predictabilities of TA traits in wild tomato population. Seven scenarios of different number of wild tomato accessions together with all cultivated accessions assigned in the training set and test on the remaining wild accessions were investigated (Scenario A). Seven scenarios of different number of wild tomato accessions together with the same number of cultivated accessions in the training set and test on the remaining accessions (Scenario B). The values of column names indicated the number of wild tomato accessions used to train the GS model. Genomic selection predictabilities evaluated using the Pearson correlation coefficient between predicted and measured values in testing set, i.e. the remaining tomato accessions, in cross-validations.

**Supplementary Table 10.** Genomic selection predictabilities of CD traits using classification-based models. Genomic selection predictabilities evaluated using the AUC and accuracy coefficients between predicted and measured values in testing set in cross-validations.
